# Changes in serum calcium, magnesium and inorganic phosphorus levels during different phases of the menstrual cycle

**DOI:** 10.4103/0974-1208.44115

**Published:** 2008

**Authors:** Puja Dullo, Neeraj Vedi

**Affiliations:** Department of Physiology, Govt. Medical College, Kota (Rajasthan), India

**Keywords:** Calcium magnesium, inorganic phosphorus, menstrual cycle

## Abstract

**Background:**

The menstrual cycle is a complex process involving the interaction of the hypothalamus, the anterior pituitary, the ovaries and the uterus. The hormonal changes occurring during this cyclic process not only affect oocyte maturation and the endometrial and vaginal environments but can also have an effect on a number of other physiological and biochemical phenomena.

**Aim and Method:**

We investigated the changes in serum calcium, magnesium and inorganic phosphorus levels during different phases of the menstrual cycle in fifty healthy young women. We found subtle but significant variations in these levels in the menstrual, follicular and luteal phases.

**Result:**

The serum calcium level was highest in the follicular phase whereas the serum magnesium level was lowest in the follicular phase; the serum inorganic phosphorus levels was highest in the menstrual phase.

**Conclusion:**

These variations could be due to the impact of the changing estrogen and progesterone secretion on the parathyroid glands.

## INTRODUCTION

The menstrual cycle is unique to female human beings and a few nonhuman primates. It results from a complex interaction between the hypothalamus, the anterior pituitary gland, the ovaries and the uterus. Hormonal changes during this cyclic process result in the ovulation of a mature oocyte from the ovary into the endometrium which is favorable for the implantation of the fertilized ovum.[1]

The average menstrual cycle of 28 days (23–29 days) is divided into three phases.[2] The first phase, an estrogen-dominated phase, lasts up to the time of ovulation, during which there is an increase of 1–5 mm in the endometrium. This phase is known as the proliferative phase or the follicular phase.[3] The second phase, the secretory or luteal phase, is due to an increase in progesterone secretion causing a coiling of the endometrial vessels and a thickening of the endometrium. In the last phase, the menstrual phase, there is a decrease in all the ovarian hormones which, in turn, decreases the production of all anterior pituitary reproductive hormones. This results in the shedding off of the superficial part of the endometrium due to a vasospasm produced possibly by locally released prostaglandins.[4–6]

The cyclic hormonal changes can affect a variety of physiological and biochemical processes. There are very few reports on the changes in serum calcium, magnesium and inorganic phosphorus levels in various phases of the menstrual cycle in otherwise healthy women. It has, however, been reported that estrogen induces hypercalcemia through the action of the parathyroid gland.[7] Withdrawal of estrogen is reported to cause a significant loss of bone calcium.[8] It was observed that an increase in the basal metabolic rate and oxygen consumption during the luteal phase was associated with increased carbohydrate utilization. This elevated metabolism requires magnesium ions and oxidative enzymes which were found to be increased significantly during the luteal phase.[9] It has also been found that the phosphate concentration falls more rapidly than the calcium rise after parathyroid hormone administration.[7] This decline in phosphate concentration is caused by a strong effect of parathyroid hormone on the kidney, causing renal phosphate excretion.[10] Pandya *et al.* have reported that serum inorganic phosphorus levels were higher in the menstrual phase as compared to the other phases.[11] These ions are regulated by various hormones over and above sex hormones.

Our aim was to assess the changes in the levels of serum calcium, magnesium and inorganic phosphorus during the different phases of the menstrual cycle in young healthy women. We also sought to assess the ratio of Ca^2+^/Mg^2+^ to help prevent i) premenstrual syndrome (PMS), ii) general disorders during pregnancy, *e.g*., preeclampsia/eclampsia, menopausal cardiovascular and bone problems (loss and susceptibility to fracture) and iii) the incidence of pathophysiology after contraceptive use as specified by Seelig.[12]

## MATERIALS AND METHODS

The study was conducted on 50 healthy unmarried females in the age group of 17–28 years. The clinical history of the subjects was noted and different phases of the menstrual cycle (menstrual, follicular and luteal phases) were determined by a detailed menstrual history. A thorough clinical check-up was done to exclude subjects suffering from neuropsychiatric disorders or any other illness affecting the menstrual cycle. Our subjects suffered from premenstrual syndrome and were asked to measure their daily oral temperature immediately after waking up in the morning, before taking any hot/cold drink, during the course of the study.

Each subject was explained the aim and the method of the test to eliminate fear and apprehension. Three milliliters of venous blood were drawn between 8 a.m. and 9 a.m. during each phase of the menstrual cycle. Blood was drawn within the first two days of the cycle during the menstrual phase, within the eighth to fourteenth day during the follicular phase and after the 22^nd^ day during the luteal phase until the next cycle began. Each sample was analyzed for levels of:

- Serum calcium[13]
- Serum magnesium[14]
- Serum inorganic phosphorus[15]

Statistical analysis of the three sets of data (menstrual, follicular and luteal phases) for each analysis was carried out by ANOVA Test.

## RESULTS

The mean ± SD age of the subjects was 21.5 ± 1.5 years (17–28 years). The duration of the menstrual cycle varied from 28 to 35 days with a mean ± SD of 29.03 ± 0.98 days. The duration of the menstrual phase varied from 2 to 6 days.

The average fall in the basal body temperature at the time of ovulation was 0.6°F (0.4–0.7°F). During the luteal phase, the temperature rose by 0.5 to 1.0°F.

Values of serum calcium, magnesium and inorganic phosphorus levels in different phases of the menstrual cycle are shown in Table 1. In the menstrual and follicular phases, the serum calcium levels varied from 8.3 to 10.5 mg/dL (9.52 ± 0.55 mg/dL) and from 9.3 to 11.0 mg/dL (10.08 ± 0.48 mg/dL) respectively. During the luteal phase, serum calcium levels varied from 8.4 to 10.6 mg/dL (9.29 ± 0.52 mg/dL). Upon analysis by ANOVA with the help of the XL-STP version of statistical software, serum calcium levels were found to be significantly higher during the follicular phase than in the other two phases. However, none of the values was outside the normal range. Serum magnesium levels were significantly lower in the follicular phase than in the other two phases. In the menstrual, follicular and luteal phases, serum magnesium levels varied from 1.5 to 2.7 mg/dL (2.01±0.26 mg/dL), from 1.3 to 2.5 mg/dL (1.76 ± 0.3 mg/dL) and from 1.7 to 2.8 mg/dL (2.18 ± 0.27 mg/dL) respectively.

**Table 1:** Serum calcium, magnesium and inorganic phosphorus (mg/dL) levels in the different phases of the menstrual cycle in healthy women

	Mean ± SD (mg/dL)		
	
	Serum calcium	Serum magnesium phosphorus	Serum inorganic
Menstrual phase	9.52 ± 0.55	2.01 ± 0.26	4.34 ± 0.44* * *
Follicular phase	10.08 ± 0.48*	1.76 ± 0.31**	3.83 ± 0.47
Luteal phase	9.29 ± 0.52	2.18 ± 0.27	3.43 ± 0.51

n = 50

* Significantly higher as compared to the other two (*P* < 0.05)

** Significantly lower as compared to the other two (*P* < 0.05);

*** Significantly higher as compared to the other two (*P* < 0.05)

Serum inorganic phosphorus levels decreased progressively and significantly from the menstrual phase to the follicular phase and from the follicular phase to the luteal phase.

Table 2 shows the ratio of serum calcium/magnesium levels in the three phases of the menstrual cycle. The ratio was the lowest in the menstrual phase and the highest in the follicular phase.

**Table 2:** Ratios of serum calcium (mg/dL) / serum magnesium (mg/dL) in different phases of menstrual cycle

	Mean ± SD		
	
	Menstrual phase	Follicular phase	Follicular phase
Serum calcium/Magnesium ratio	4.74 ± 0.58	5.74 ± 0.27*	4.26 ± 0.37

* Significantly higher as compared to other two (*P* < 0.001)

## DISCUSSION

The menstrual cycle is the most extensively studied rhythm in women. The hormonal changes during the normal menstrual cycle are well established and these hormonal changes are commonly associated with fluctuations in the state of physiological functions and subjective feeling in women. An extensive literature search has revealed very scanty data for the changes in serum calcium, magnesium and inorganic phosphorus levels in the various phases of the menstrual cycle. Changes in these ions are, however, reported to be mainly due to changes in the hormonal levels during the different phases of the menstrual cycle.[8–10]

In the present study, the mean serum calcium levels increased by 5.61% in the follicular phase as compared to the menstrual phase and decreased by 7.86% in the luteal phase. Earlier research shows that the increase in serum calcium levels during the follicular and ovulatory phases could be due to the effect of estrogen on the parathyroid glands.[16] Reportedly, the higher levels of progesterone compared to estrogen during the luteal phase could be responsible for these low serum calcium levels.[17] Our results are in agreement with these reports.

We have found a decrease of 12.42% in serum magnesium levels in the follicular phase as compared to the menstrual phase and an increase of 19.45% in the luteal phase as compared to the follicular phase. Thus, the levels of serum magnesium were highest during the luteal phase and lowest during the follicular phase. These results are in agreement with the observations of Pandya *et al.*[11] The raised estrogen levels could possibly be acting on the parathyroid gland, due to which serum magnesium levels dropped during the ovulatory phase as reported by Pitkin *et al.* Pitkin and co-workers further stated that clinical hyperparathyroidism could deplete the body stores of magnesium.[16] It has been reported that magnesium ions and oxidative enzymes are needed for carbohydrate utilization which increases significantly during the luteal phase.[18]

Increased serum calcium levels during the ovulatory phase may also contribute to the decreased magnesium levels by exerting an effect on the cell permeability.[19,20] It is well known that low magnesium levels result in the constriction of cerebral and abdominal blood vessels.[21] It is, thus, also possible that the water retention (bloating) that occurs during the luteal phase results, in part, from a slight increase in the constriction of the renal arterial vessels. It has been demonstrated during studies on cerebral peripheral blood vessels as well as on *in vitro* umbilical-placental blood vessels, that elevated calcium/magnesium ratios induce spasm.[22]

The fact that the increase in Ca^2+^/Mg^2+^ ratio coincides with the peak of estrogen and with the increase in progesterone would confirm that this effect is present throughout the menstrual period. It is also suggested that this ratio may be related to the PMS complaints that some women have during this period. Elevated Ca^2+^/Mg^2+^ ratios are also associated with the onset of migraine and tension headaches.[23]

In the present study, the serum inorganic phosphorus levels decreased by 11.66 and 20.88% in the follicular and luteal phases respectively as compared to the menstrual phase. This pattern is consistent with an earlier report in which serum inorganic phosphorus levels were found to be higher during the menstrual phase than in the other two phases.[11] The decrease in inorganic phosphorus levels with increased follicular Ca^2+^/Mg^2+^ ratios in the luteal phase as compared to the menstrual phase could be due to estrogen as reported earlier.[21] The present study also compares well with an earlier observation that high estrogen production can lead to a decrease in serum inorganic phosphorus levels.[24]

## CONCLUSION

This study enabled us to measure the serum calcium, magnesium and inorganic phosphorus levels as well as the Ca^2+^/Mg^2+^ ratios in females who suffered from PMS. We were able to reduce varied PMS symptoms in them with the use of magnesium infusion or its salts along with Vitamin D during the second week of the luteal phase as suggested by Mauskop *et al.*[25]
